# Temas de contenido y voces influyentes dentro de la oposición a las vacunas en Twitter, 2019^*^

**DOI:** 10.26633/RPSP.2021.54

**Published:** 2021-05-12

**Authors:** Erika Bonnevie, Jaclyn Goldbarg, Allison K. Gallegos-Jeffry, Sarah D. Rosenberg, Ellen Wartella, Joe Smyser

**Affiliations:** 1 The Public Good Projects Alexandria Estados Unidos de América The Public Good Projects, Alexandria, Estados Unidos de América.; 2 Northwestern School of Communication Evanston Estados Unidos de América Northwestern School of Communication, Evanston, Estados Unidos de América.

**Keywords:** Información, redes sociales, salud pública, vacunas

## Abstract

**Objetivo.:**

Informar sobre la oposición a las vacunas y la información errónea fomentadas en Twitter, destacando las cuentas de Twitter que dirigen estas conversaciones.

**Métodos.:**

Utilizamos el aprendizaje automático supervisado para codificar todos los mensajes publicados en Twitter. En primer lugar, identificamos manualmente los códigos y los temas mediante un enfoque teórico fundamentado y, a continuación, los aplicamos a todo el conjunto de datos de forma algorítmica. Identificamos a los 50 autores más importantes un mes tras otro para determinar las fuentes influyentes de información relacionadas con la oposición a las vacunas.

**Resultados.:**

El período de recopilación de datos fue del 1 de junio al 1 de diciembre del 2019, lo que dio lugar a 356 594 mensajes opuestos a las vacunas. Un total de 129 autores de Twitter reunieron los criterios de autor principal durante al menos un mes. Los autores principales fueron responsables del 59,5% de los mensajes opuestos a las vacunas y detectamos diez temas de conversación. Los temas se distribuyeron de forma similar entre los autores principales y todos los demás autores que declararon su oposición a las vacunas. Los autores principales parecían estar muy coordinados en su promoción de la información errónea sobre cada tema.

**Conclusiones.:**

La salud pública se ha esforzado por responder a la información errónea sobre las vacunas. Los resultados indican que las fuentes de información errónea sobre las vacunas no son tan heterogéneas ni están tan distribuidas como podría parecer a primera vista, dado el volumen de mensajes. Existen fuentes identificables de información errónea, lo que puede ayudar a contrarrestar los mensajes y a fortalecer la vigilancia de la salud pública.

## Abstract (en)

**Objectives.:**

To report on vaccine opposition and misinformation promoted on Twitter, highlighting Twitter accounts that drive conversation.

**Methods.:**

We used supervised machine learning to code all Twitter posts. We first identified codes and themes manually by using a grounded theoretical approach and then applied them to the full data set algorithmically. We identified the top 50 authors month-over-month to determine influential sources of information related to vaccine opposition.

**Results.:**

The data collection period was June 1 to December 1, 2019, resulting in 356 594 mentions of vaccine opposition. A total of 129 Twitter authors met the qualification of a top author in at least 1 month. Top authors were responsible for 59.5% of vaccine-opposition messages. We identified 10 conversation themes. Themes were similarly distributed across top authors and all other authors mentioning vaccine opposition. Top authors appeared to be highly coordinated in their promotion of misinformation within themes.

**Conclusions.:**

Public health has struggled to respond to vaccine misinformation. Results indicate that sources of vaccine misinformation are not as heterogeneous or distributed as it may first appear given the volume of messages. There are identifiable upstream sources of misinformation, which may aid in countermessaging and public health surveillance.

La oposición a las vacunas es una amenaza para la salud mundial (1), y los medios digitales y sociales son una de las principales fuentes de información errónea y un medio para organizar la oposición a las vacunas (2,3). La información errónea ha alcanzado un nivel crítico, con comunidades a favor y en contra de las vacunas cada vez más polarizadas (4). Los mensajes “anti” están aumentando en comunidades que parecen no estar afectadas por las estrategias tradicionales de promoción de la salud y la información científica (5). En el año 2000, se declaró la erradicación del en Estados Unidos gracias a una eficaz campaña de vacunación; sin embargo, en el 2019, los Centros para el Control y la Prevención de Enfermedades anunciaron 1282 casos confirmados de sarampión, el número más elevado desde 1992 (6).

La oposición a las vacunas también tiene implicaciones políticas: docenas de proyectos de ley estatales han intentado suplantar esta práctica establecida de salud de la población dando prioridad a las libertades personales y apelando a la ideología, en lugar de a la evidencia (7). La información errónea erosiona la confianza en la ciencia y en las autoridades de salud pública, y se asocia con una disminución de las tasas de vacunación, lo que supone el riesgo de nuevos brotes y casos de enfermedades prevenibles por vacunación (8). Esto también tiene implicaciones económicas: el tratamiento de los brotes de sarampión cuesta aproximadamente $32 000 por caso (9) y, en el 2017, el costo informado de tratar un caso de tétanos infantil fue de más de $800 000 (10). A pesar de la amenaza establecida y en evolución que supone la oposición a las vacunas para la salud pública, en Estados Unidos no ha habido un esfuerzo sistemático y sostenido para identificarla, rastrearla e informar sobre ella de forma regular.

En el 2019, The Public Good Projects, una organización de salud pública sin fines de lucro, puso en marcha un proyecto para detectar y rastrear las comunicaciones relacionadas con las vacunas en los medios digitales y sociales. Este estudio examina el discurso en Twitter, dado que esa plataforma es una fuente principal de información errónea en línea sobre las vacunas (11,12). Los objetivos de este estudio fueron: 1) determinar el volumen de las conversaciones en torno a la oposición a las vacunas, 2) examinar temas específicos en las conversaciones sobre la oposición a las vacunas con un énfasis en la información errónea relacionada con las vacunas, y 3) identificar las cuentas que impulsan la oposición a las vacunas. Se compararon los temas de contenido empleados por las cuentas influyentes sobre la oposición a las vacunas con los temas generales del discurso de la oposición a las vacunas para determinar los marcos de los mensajes que los autores principales usan para dirigir la conversación.

## MATERIALES Y MÉTODOS

Se obtuvieron los datos por medio de una asociación con una plataforma de monitorización de medios de comunicación que recogió el 100% de los tuits y retuits de Twitter disponibles públicamente que contenían palabras clave identificadas por The Public Good Projects. El proceso inicial de recopilación de datos se basó en una extensa consulta de búsqueda de palabras clave utilizando operadores booleanos en inglés para detectar la información relacionada con las conversaciones sobre vacunación en Twitter en Estados Unidos desde el 1 de junio hasta el 1 de diciembre del 2019. Las palabras clave se seleccionaron a partir de una revisión de la bibliografía científica, gris y blanca publicada anteriormente y de determinaciones deductivas basadas en la familiaridad con la conversación en línea sobre vacunas.

La recopilación inicial de datos siguió dos procesos: las palabras clave podían ser “independientes” o “concurrentes”. Las palabras clave independientes funcionan de manera que cualquier mención de una palabra específica haría que se recopile ese mensaje. La consulta inicial constaba de 129 palabras aisladas y 129 equivalentes de etiquetas o *hashtags*. Los términos también podían ser concurrentes, lo que significa que si estaban presentes dos términos se recopilaría ese mensaje. Se recogieron formas abreviadas del término "vacunación" en inglés si también incluían alguna afección de salud tratada por las vacunas o términos referidos en el discurso sobre las vacunas. La consulta de búsqueda concurrente consistió en 333 palabras relacionadas con estas afecciones de salud o con las vacunas y sus equivalentes en etiquetas, emparejados con tres términos abreviados de vacunas y sus equivalentes en etiquetas. Se emplearon 60 términos de exclusión para excluir los contenidos relacionados con la vacunación de animales o las instrucciones de medicación.

### Identificar la oposición a las vacunas

**FIGURA 1.**
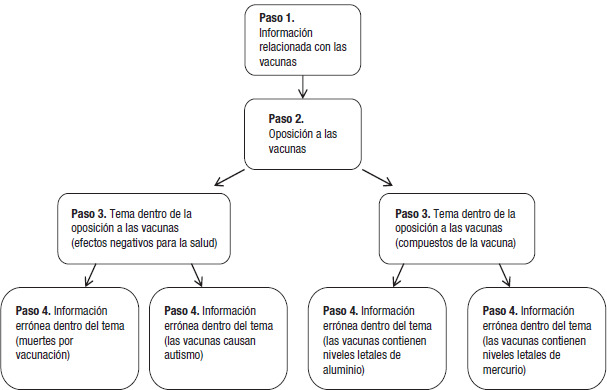
Proceso de recopilación y codificación de datos de Twitter para su análisis

Se obtuvieron datos de Twitter de manera continua durante todo el período de recopilación de datos. Con la recopilación de datos en curso, se seleccionó una muestra aleatoria de 1000 tuits de la muestra total de conversaciones relacionadas con las vacunas (0,9% de los datos recopilados en ese momento, en consonancia con las investigaciones que realizan análisis similares) (13) y se codificaron de forma manual manualmente para identificar los mensajes contrarios a las vacunas (paso 2 en la figura 1). En este proceso, los retuits no se codificaron manualmente, dado que a menudo son idénticos al tuit original, y los analistas se centraron en codificar el mayor número posible de mensajes únicos. Estos mensajes son la muestra total, que contenía todos los mensajes que hacían referencia a las vacunas, ya sean positivos, neutros o en oposición. Los mensajes en los que se hacía referencia a cierta reticencia frente a las vacunas (es decir, los correspondientes a personas que no se vacunan por falta de acceso o que se vacunan, pero tienen dudas) no se consideraron opuestos a las vacunas.

Estos mensajes publicados generaron una lista adicional de palabras clave específicas sobre la oposición a las vacunas, que se añadieron a la consulta de palabras clave completa que generó la muestra total, lo que permitió identificar y analizar los mensajes por separado de oposición a las vacunas. Todos los análisis de este estudio se realizaron sobre los mensajes que contenían términos relacionados con la oposición a las vacunas.

### Creación de temas

A continuación, se clasificaron los mensajes opuestos a las vacunas por temas. Mediante un proceso interpretativo de cinco pasos, dos codificadores (E.B. y S.D.R.) codificaron manualmente 1000 mensajes publicados seleccionados al azar (paso 3 en la figura 1) (14). Se codificaron aproximadamente 200 mensajes de forma cruzada entre los analistas. Las discrepancias se reexaminaron hasta alcanzar un acuerdo en más de 90% de los mensajes. Se crearon, compararon y combinaron los temas hasta que se alcanzó la saturación de datos, definida como un tema que comprende menos del 1% de la conversación. En este estudio, el 74,8% de los datos relativos a la oposición a las vacunas se codificaron en un tema. A cada tema se le asignó una lista única de palabras clave que identificaban una publicación que cumplía el criterio de ese tema. Para comprobar la validez de las palabras clave de cada tema, estas se convirtieron en consultas de términos, como se ha descrito anteriormente. Revisamos 100 mensajes seleccionados al azar y clasificados automáticamente para cada tema. Si el 90% de los mensajes clasificados automáticamente estaban codificados de manera correcta, se aprobaba la consulta de palabras clave de ese tema y se aplicaba a la muestra total. Las aplicaciones de la codificación automática supervisada para el análisis cualitativo han sido exploradas como una forma práctica de aplicar las enseñanzas obtenidas de los grandes conjuntos de datos a la salud pública (13,15).

Para identificar la información errónea promovida dentro de los temas relacionados con la oposición a las vacunas, los analistas revisaron 200 mensajes que registraron el mayor volumen de interacción dentro de cada tema (paso 4 en la figura 1). La información errónea se organizó en categorías, y cada categoría se definió con palabras clave únicas. Estas palabras clave permitían etiquetar automáticamente todos los mensajes del tema si contenían una categoría de información errónea. Los analistas verificaron manualmente los 200 mensajes principales dentro de cada categoría de información errónea, y se modificaron las palabras clave para garantizar que al menos el 90% de los mensajes estuvieran etiquetados con la categoría correcta de información errónea. En la definición operativa de “información errónea sobre vacunas” se tuvo en cuenta toda información que contrastara con la Oficina de Seguridad de la Inmunización, de los Centros de Control y Prevención de Enfermedades (16).

### Autores principales

Al igual que en otros estudios en los que se examinan los mensajes de Twitter en busca de información relacionada con las vacunas (17,18), el presente estudio hizo uso de los metadatos que acompañan a los mensajes para realizar análisis de redes sociales. Se clasificaron las cuentas que publicaban mensajes por el número de interacciones recibidas para determinar cuáles eran las cuentas que tenían más influencia en la conversación sobre la oposición a las vacunas (denominadas “autores principales”). Las interacciones consistieron en un “me gusta”, un comentario o bien en compartir una publicación. Los analistas identificaron a los 50 autores principales de cada mes. Definir la interacción de esta manera permitió descubrir las cuentas con interacciones más frecuentes, los mensajes específicos que recibieron más interacciones y los temas más abordados en esos mensajes. Una investigación anterior también examinó a los 50 autores principales de Twitter como una forma de medir las tendencias (19). Los autores principales fueron examinados manualmente para asegurarse de que estaban promoviendo la oposición a las vacunas, en lugar de burlarse o informar sobre la oposición a las vacunas. Los resultados comparan la conversación de los autores principales con la conversación general de la oposición a las vacunas con los autores principales eliminados (“autores principales” frente a “autores no principales”). Se utilizó la prueba de la χ^2^ para determinar las diferencias estadísticamente significativas entre los autores principales y los no principales para cada tema.

## RESULTADOS

Del 1 de junio al 1 de diciembre del 2019, se recopilaron 356 594 mensajes en Twitter que mencionaban la oposición a las vacunas. Se identificaron 129 cuentas de Twitter como autores principales en un período mínimo de 1 a 6 meses, que dieron lugar a un total de 212 018 interacciones y 772,9 millones de impresiones potenciales (el número de seguidores del autor original más los seguidores de personas que compartieron su contenido). De esas 129 cuentas, 15 fueron autores principales durante al menos 5 meses, durante los cuales generaron 124 243 interacciones, lo que supone el 58,6% de las 212 019 interacciones con los contenidos de los autores principales.

Se identificaron diez temas dentro de los mensajes sobre la oposición a las vacunas, y los cinco temas más importantes representaron cada uno más del 10% de las menciones (cuadro 1).

Las repercusiones negativas para la salud se manifestaron en el 55,4% de las menciones de los autores principales y en el 49,2% de la oposición general (autores no principales). Dentro de este tema, la información errónea en torno a las muertes atribuibles a las vacunas y al autismo causado por las vacunas estuvo presente en el 66,5% y el 43,8%, respectivamente, de los mensajes de los autores principales. Dentro de la oposición general, las muertes se mencionaron en el 14,5% de los mensajes y el autismo en el 26,3%. En cuanto a las referencias a la muerte, los autores principales compartieron predominantemente un artículo científico que citaba las muertes notificadas al sistema de notificación de reacciones adversas a las vacunas entre el 1997 y el 2013 para afirmar que las vacunas causan mortalidad infantil (20). Otra información errónea relacionada con los efectos en la salud incluía las asociaciones entre las vacunas y la parálisis (5,9% de los autores principales; 0,5% de oposición general) y convulsiones (5,7% de los autores principales; 0,8% de oposición general).

**CUADRO 1.** Proporción de temas de conversación sobre la oposición a las vacunas en Twitter, del 1 de junio al 1 de diciembre del 2019

Tema	Autores principales: oposición a las vacunas^a,b^ (%, n)	No. autores principales^b,c^ (%, n)	Valor de *P*
Efectos negativos sobre la salud atribuidos a la vacunación	55,4 (117 530)	49,2 (71 167)	<0,001
Industrias farmacéuticas	16,9 (35 821)	18,9 (27 346)	<0,001
Investigación y ensayos clínicos	15,5 (32 819)	5,6 (8097)	<0,001
Normas y políticas	15,0 (31 723)	17,7 (25 621)	<0,001
Compuestos de las vacunas	13,8 (29 281)	17,2 (24 858)	<0,001
Familia	7,3 (15 508)	7,4 (10 628)	0,68
Prevalencia y brotes de enfermedades	5,1 (10 885)	3,2 (4579)	<0,001
Escuela	3,6 (7733)	2,8 (3997)	<0,001
Religión	3,2 (6884)	2,3 (3343)	<0,001
Alternativas naturales	0,9 (1953)	1,6 (2287)	<0,001

a Calculado a partir de todos los mensajes e interacciones relativas al contenido de los autores principales (n = 212 018 de un total de 356 594 mensajes).

b Los porcentajes pueden sumar más del 100%, ya que un mensaje puede codificarse en varios temas.

c Calculado a partir de todos los mensajes e interacciones relacionadas con la oposición a las vacunas o las dudas sobre la vacunación, menos los autores principales (n = 144 576 de un total de 356 594 mensajes).

Las menciones a la industria farmacéutica aparecieron en el 16,9% de los mensajes de los autores principales y en el 18,9% de la oposición general. La mayoría de las veces, las vacunas se enmarcan en una conspiración de las “grandes farmacéuticas” para aumentar los ingresos por ventas. Merck fue mencionada en el 58,1% de los mensajes de los autores principales, en comparación con el 38,7% de la oposición general, debido a su fabricación de la vacuna Gardasil.

Las políticas y los debates políticos relacionados con la vacunación vinieron a continuación, en un 15,0% de las conversaciones de los autores principales y en un 17,7% de la oposición general. Los mensajes relativos a este tema se centraron predominantemente en la ley nacional de lesiones por vacunas en la infancia, que eliminó la posible responsabilidad financiera de los fabricantes de vacunas en las demandas por lesiones (27,2% de los autores principales; 6,6% de la oposición general) y el proyecto de ley 276 del Senado de California que endurece las exenciones de las vacunas (23,9% de los autores principales; 17,5% de oposición general). En el discurso político sobre las vacunas se menciona con frecuencia el papel del gobierno en las reclamaciones por lesiones causadas por vacunas y las acusaciones de que el gobierno oculta deliberadamente los efectos secundarios negativos de las vacunas.

Los compuestos de las vacunas constituyeron el 13,8% de las conversaciones de los autores principales y el 17,2% de las de la oposición general, con mensajes que citaban metales pesados o compuestos mencionados en los prospectos de las vacunas. El aluminio fue el compuesto referenciado con más frecuencia, en el 44,5% de los mensajes de los autores principales y en el 6,4% de los de la oposición general, seguidos del mercurio (34,1% de los autores principales; 6,9% de la oposición general) y el tejido fetal abortado (9,3% de los autores principales; 2,6% de la oposición general).

La investigación sobre las vacunas apareció en el 15,5% de los mensajes de los autores principales y en el 5,6% de los de la oposición general. La mayoría de los mensajes criticaban la investigación sobre las vacunas o las instituciones que la llevaban a cabo, o promovían la pseudociencia como un hecho. Los estudios más referenciados estaban relacionados con la vacuna contra el virus del papiloma humano y su asociación con efectos negativos en la salud después de la vacunación (21). Un artículo compartido comúnmente fue retirado en el 2019 y ahora se encuentra en sitios web de oposición a las vacunas (29,0% de los autores principales; 8,9% de la oposición general) (22,23). A esto le siguieron las investigaciones sobre la vacuna antigripal, destacando los estudios que mostraban asociaciones con otras infecciones respiratorias, insuficiencia renal y supresión de la respuesta inmunitaria (20,0% de los autores principales; 7,3% de la oposición general) (24-26).

Cinco de los temas identificados representaron aproximadamente el 7% o menos de la conversación total.

La prevalencia de enfermedades se centró en los brotes de sarampión, ya que el 83,2% de los mensajes de los autores principales y el 17,4% de los mensajes de la oposición general mencionaron el sarampión o la vacuna contra el sarampión, las paperas y la rubéola. Los opositores a las vacunas citaron con frecuencia comentarios sobre epidemias provocadas por las vacunas, como el poliovirus derivado de la vacuna, para sugerir los peligros de las vacunas (19,5% de los autores principales; 3,8% de la oposición general) (27).

Los familiares solían mencionar a personas que creían haber experimentado efectos negativos en la salud atribuibles a la vacunación, a menudo un padre que hablaba de un episodio de reacción adversa a una vacuna de su hijo (70,7% de los autores principales; 57,2% de la oposición general).

La conversación en torno a las escuelas se centró en las políticas relacionadas con la vacunación obligatoria para la inscripción.

La religión incluyó referencias a cualquier religión y la mayoría de las veces se debatía acerca de exenciones religiosas a las vacunas obligatorias (46,7% de los autores principales; 44,5% de la oposición general).

Las alternativas naturales a las vacunas incluían información errónea sobre el uso de alternativas homeopáticas a la vacunación y la “desintoxicación de las vacunas”.

## DISCUSIÓN

Este estudio demostró que los principales argumentos utilizados por los opositores a las vacunas procedían de un puñado de cuentas. Un total de 129 cuentas de Twitter parecían estar fomentando más de la mitad de toda la conversación relativa a la oposición a las vacunas, y 15 cuentas parecían ser muy influyentes, pues generaban la mayoría de las interacciones a los mensajes de los autores principales. Cuando se comparan los mensajes de los autores principales con otros mensajes, los temas de información errónea son similares. Aunque hubo diferencias estadísticamente significativas en las proporciones de la mayoría de los temas, esto puede ser atribuible al tamaño de la muestra; cuando se clasificaron los temas por su uso, los temas más comunes utilizados por los autores principales y por todos los demás autores eran casi idénticos.

Cuando examinamos los temas de conversación específicos, los autores principales promovieron información errónea similar dentro de cada tema. Por ejemplo, dentro de la conversación sobre las repercusiones negativas para la salud, las referencias a las muertes y al autismo se mencionaron en el 67% y el 44% de los mensajes de los autores principales, respectivamente. En los mensajes de los autores no principales, estas dos consecuencias se mencionaron en aproximadamente el 15% de los mensajes. En todos los temas, los resultados mostraron cómo los opositores a las vacunas pueden manipular los hechos y sus fuentes. Verificar cada una de las afirmaciones hechas por un opositor a las vacunas puede ser un reto, incluso para los investigadores de salud pública con experiencia, sobre todo teniendo en cuenta la cantidad, la variedad y la naturaleza a menudo engañosa de los mensajes. Por ejemplo, la información extraída del sistema de notificación de reacciones adversas a las vacunas, una base de datos creada por los organismos federales de salud para vigilar las reacciones a las vacunas, es utilizada por los opositores a las vacunas como "prueba" de que el gobierno admite que las vacunas causan mortalidad infantil. De ese modo se pierde un contexto de importancia crítica, como el hecho de que una reacción adversa puede notificarse incluso si es incierto o poco probable que una vacuna lo haya causado o el papel de la significación estadística o el sesgo de notificación en epidemiología. La información errónea es una cuestión compleja que implica no solo lo que se dice, sino también la intención que hay detrás de ello.

El hallazgo de que los autores principales comparten la misma información errónea sugiere que los opositores a las vacunas dependen de comunidades altamente interconectadas, impulsadas por líderes que manejan relatos específicos (28,29). Es muy probable que los opositores influyentes a las vacunas seleccionen sus mensajes en función de la receptividad que tengan. Por el contrario, la salud pública sigue repitiendo las mismas recomendaciones de vacunación de la misma manera, a pesar de que las investigaciones demuestran que esos mensajes llegan solo a aquellos que es poco probable que duden de las vacunas (2,4). La comunidad de salud pública debe pensar críticamente y centrar los mensajes en los temas que reciben la mayor atención entre quienes es probable que tengan reticencia frente a la vacunación.

El estudio sugiere que los temas principales divulgados por la oposición a las vacunas no solo se pueden descubrir, sino que también se pueden cuantificar; solo se abordan unos cuantos en cualquier momento dado. Esto concuerda con la investigación que señala que la mayoría de los anuncios de Facebook que se oponen a la vacunación fueron financiados por dos grupos (30). Si esos grupos se vigilan pasivamente, como sugieren otros investigadores, la salud pública puede ser capaz de contrarrestar la influencia cada vez mayor de la oposición a las vacunas mediante la identificación rápida de los temas de conversación y su neutralización (4).

### Limitaciones

El estudio tuvo algunas limitaciones. Se recopilaron los tuits que contenían las palabras clave identificadas (apéndice B). No se recopilaron los tuits sobre vacunas que no contenían estos términos. Es posible que los mensajes se hayan codificado mal, sobre todo los que se refieren sarcásticamente a la oposición a las vacunas. Los analistas comprobaron manualmente cada tema para garantizar una fidelidad de al menos el 90% y modificaron las palabras clave para captar el sarcasmo cuando fuera posible. Además, las interacciones y los temas de discusión compartidos se utilizaron como mediciones de la influencia, aunque es probable que haya otros medios inexplorados para cuantificar la influencia de las personas en las redes sociales. Además, es posible que las interacciones con los mensajes de los autores principales criticaran la oposición a las vacunas, en lugar de apoyarla. Para abordar las limitaciones, durante el estudio se ensayó y comprobó la metodología de codificación automática y se consultaron investigaciones anteriores sobre el análisis automático de la percepción relativa a la oposición a las vacunas en Twitter (31,32).

Aunque está fuera del alcance de este estudio, debería examinarse el efecto de la estacionalidad en la oposición a las vacunas. El período de recopilación de datos abarcó la temporada de regreso a la escuela, la temporada de gripe y el ciclo legislativo. La estacionalidad fue probablemente un factor que contribuyó a la información errónea. Además, variables como la hora o el día de la semana podrían ser útiles para entender la difusión del mensaje.

### Implicaciones para la salud pública

Los resultados pusieron de manifiesto la información errónea común relacionada con las vacunas que utilizan quienes se oponen a ellas. Seguirá siendo difícil para la salud pública contrarrestar eficazmente la oposición a las vacunas sin una mayor comprensión de los actores y el discurso de la oposición. También es importante señalar que, aunque el estudio examinó la oposición a las vacunas de forma colectiva, las opiniones a favor y en contra de la vacunación están mejor representadas como un espectro, no como estados distintos (33). Otras investigaciones deberían dividir los públicos que pueden ser susceptibles a mensajes específicos destacados en el estudio. De este modo, los investigadores pueden identificar formas de utilizar y compartir datos retrospectivos, en tiempo real y predictivos de los medios de comunicación para crear mensajes que lleguen de forma eficaz y rápida a las personas que son reticentes a la vacunación.

## Protección de los participantes.

En este estudio no participaron personas. Se solicitó la aprobación de la junta de revisión institucional a IntegReview, y se consideró que el estudio estaba exento de revisión.

## Declaración.

Las opiniones expresadas en este manuscrito son responsabilidad del autor y no reflejan necesariamente los criterios ni la política de la *RPSP/PAJPH* y/o de la OPS.
